# Accurate Traffic Flow Prediction in Heterogeneous Vehicular Networks in an Intelligent Transport System Using a Supervised Non-Parametric Classifier

**DOI:** 10.3390/s18061696

**Published:** 2018-05-24

**Authors:** Hesham El-Sayed, Sharmi Sankar, Yousef-Awwad Daraghmi, Prayag Tiwari, Ekarat Rattagan, Manoranjan Mohanty, Deepak Puthal, Mukesh Prasad

**Affiliations:** 1 College of Information Technology, UAE University, Al Ain 15551, United Arab Emirates; 2 Department of Information Technology, Ibri College of Applied Sciences (MoHE), Ibri 516, Sultanate of Oman; sharmisankar41@gmail.com; 3 Department of Computer Systems Engineering, Palestine Technical University, Tulkarem 007, Palestine; y.awwad@ptuk.edu.ps; 4 Department of Information Engineering, University of Padova, 35131 Padova, Italy; prayagforms@gmail.com; 5 Faculty of Information Science and Technology, Mahanakorn University of Technology, Bangkok 10530, Thailand; ekarat@mutacth.com; 6 Department of Computer Science, University of Auckland, Auckland 1010, New Zealand; m.mohanty@auckland.ac.nz; 7 School of Electrical and Data Engineering, FEIT, University of Technology Sydney, Sydney 2007, Australia; deepak.puthal@uts.edu.au; 8 Centre for Artificial Intelligence, School of Software, FEIT, University of Technology Sydney, Sydney 2007, Australia

**Keywords:** HETVNET, QoS, SVM, RBF, internet of vehicles

## Abstract

Heterogeneous vehicular networks (HETVNETs) evolve from vehicular ad hoc networks (VANETs), which allow vehicles to always be connected so as to obtain safety services within intelligent transportation systems (ITSs). The services and data provided by HETVNETs should be neither interrupted nor delayed. Therefore, Quality of Service (QoS) improvement of HETVNETs is one of the topics attracting the attention of researchers and the manufacturing community. Several methodologies and frameworks have been devised by researchers to address QoS-prediction service issues. In this paper, to improve QoS, we evaluate various traffic characteristics of HETVNETs and propose a new supervised learning model to capture knowledge on all possible traffic patterns. This model is a refinement of support vector machine (SVM) kernels with a radial basis function (RBF). The proposed model produces better results than SVMs, and outperforms other prediction methods used in a traffic context, as it has lower computational complexity and higher prediction accuracy.

## 1. Introduction

Heterogeneous vehicular networks (HETVNETs) are a subclass of ad hoc networks, and differ in characteristics from other networks as vehicles are the nodes that can compute, sense, and communicate via various access technologies, such as DSRC (Dedicated Short-Range Communications), 3G (Third Generation), UMTS (Universal Mobile Telecommunications System) etc. [1,2]. The environment is highly dynamic in nature due to the varying node positions, which causes sparse distribution of vehicles and intermittent connectivity among them, and consequently, messages are lost or delayed [3]. To ensure reliable content dissemination in HETVNETs, solutions such as name-based communication and in-network caching were proposed [4]. Each and every network node in this vehicular environment has to carry, store, and forward data with cooperative behavior. However, the “store–carry–forward” solution may cause more delay and increase overhead communication. Therefore, we need the classification and prediction of traffic to obtain information about future traffic conditions, and to adapt dissemination/routing protocol parameters based on these conditions.

Although previous studies have provided intelligent transportation systems with fundamental prediction methods, this field still lacks a classification and prediction method that can be applied to various traffic conditions, and which can produce accurate results in a short computation time. Related traffic classification and prediction methods can be categorized as parametric and non-parametric. Parametric methods have a finite number of parameters, and include linear support vector machines, Bayesian networks, neural networks, and port-based, IP-based, payload-based, behavior-based, and flow-based algorithms. Non-parametric methods have an infinite number of parameters, and include decision trees, k-means algorithms, and semi-supervised learning algorithms. Research has shown that non-parametric methods produce more accurate results than parametric methods because the infinite number of parameters assists in capturing the non-linearity of traffic flow [5].

This paper proposes a prediction model based on support vector machines (SVMs). SVMs are a non-parametric supervised machine learning technique that use associated learning algorithms to analyze data and recognize patterns [6]. SVMs are easily deployed through the mapping of non-linear data to high-dimensional feature space, and acquire a non-linear decision boundary in input space. The use of kernels twists support vector methods, and allows them to deal with other complex algorithms. Therefore, we adopted SVMs with a radial basis function (RBF) kernel for training them, as it has high performance in linear and non-linear SVMs. The contribution of this paper is a classification and prediction method that can be applied to various traffic conditions and achieve accurate results in a short computation time. The method was evaluated using a real dataset from Cambridge University (CRAWDAD, Cambridge, UK), and outperformed other methods. Although the method was tested offline, it can be used in a cloud system connected to HETVNETs. Traffic data are sent to vehicles continuously by, for example, roadside units. The routing/broadcasting protocols can utilize these data to adapt the dissemination of messages to traffic conditions, and consequently, message delay or loss can be avoided.

This paper is structured as follows: the first section presents the introduction of the paper. The second section shows the works related to the proposed model. The third section explains the theoretical background behind the proposed model to show that the model is based on a scientific framework. The fourth section shows the proposed model and how it is applied. The fifth section explains how the proposed model is accommodated to a real traffic dataset, and presents the results of using this model. Finally, the sixth section concludes the paper and outlines future directions.

## 2. Related Work

The classification of network traffic is a never-ending process as technology keeps evolving, and as such, the classification approaches and techniques need to be changed [7]. Additionally, routing protocols can overcome classical challenges such as intermittent connectivity or broadcasting storms if traffic conditions can be known in advance, which assists in the development of adaptive protocols [8]. Primarily, in order to classify traffic, a deep understanding of traffic characteristics is necessary. In this context, several fundamental classification methods, categorized as parametric and non-parametric, were used. Currently, researchers have started exploring machine learning algorithms, which reveal more promising outcomes when compared to other parametric classifiers, such as C4.5 decision trees, Bayesian networks, or neural networks [9]. Other methods, such as port-based, IP-based, payload-based, behavior-based, and flow-based algorithms, have produced promising results in various traffic situations [9]. Other studies deployed k-means algorithms from machine learning, a semi-supervised learning approach to clustering [10]. The attempt of a semi-supervised approach to clustering demonstrated better accuracy, significantly improving the categorization of real-time traffic.

Non-parametric approaches were proposed to compute correlation in the traffic dataset and predict the corresponding classes. These approaches were quoted to improve the classification performance, as they incorporated the correlation information. Research concluded that a similar approach to incorporating the correlation information can be deployed to mine and predict traffic in HETVNETs [11]. This approach to correlation information outperformed the neural network method of traffic classification [5]. Researchers have claimed that most parametric linear machine learning models are less accurate than non-parametric models [5,12,13].

The time complexity concept quantifies the computation time required to run a specific algorithm. Determining the time complexity of a classification or prediction model assists in judging whether this model can be applied online to vehicular ad hoc networks (VANETs) or not. Several studies have referred to the time complexity concept as computational demand, efficiency, or time demand, and have specified that traffic systems require fast forecasting algorithms that process data quickly enough to enable real-time decisions [5,14]. To reduce computational cost, studies have used data from limited correlated roads, instead of examining all roads. Furthermore, studies have tried to adopt efficient forecasting methods. However, most widely-used models, such as autoregression and neural network-based models, require substantial computation time [15,16]. In summary, our proposed model is a non-parametric model that has the ability to capture traffic nonlinearity and to address different traffic conditions. We should also focus on models that have a short computation time to allow real-time applications of our proposed model.

## 3. Theoretical Background

Support vector machines (SVMs) are being increasingly utilized in real-world applications, and have been labeled by researchers as a state-of-the-art tool among existing soft-computing techniques. SVMs ensure a precise perception of learning from samples, and can lead to the production of better outcomes for many practical real-world applications. The motivation behind the ease in deploying SVMs is that the mapping of non-linear data to high-dimensional feature space acquires a non-linear decision boundary in input space. In our study, input data representations were mapped into feature space by kernels.

**Figure 1:**
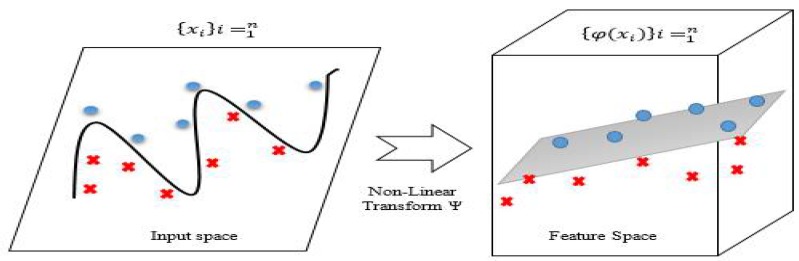
Non-linearization (Source: Introduction to Statistical Machine Learning, MIT Press, Cambridge, MA, USA, 2012 [7]).

SVMs come under the category of non-parametric classifiers, and have many types of kernels. The kernel functions are introduced in SVMs to solve classification or regression difficulties in which the data are not linearly separable [17]. The various types of SVMs with kernels are shown in Table 1, where γ, *d*, and *r* are kernel parameters. Researchers recommend choosing appropriate sensible kernel functions, and tuning related significant parameters mapping the non-linearly-separable data. The mapping of non-linearly-separable data into feature space is pictorially represented in Figure 1.

Mathematically, two critical points define a channel called a support vector, where one point exists from each class. Support vectors define the boundary between classes, and all other instances will be deleted without changing the boundary. SVMs drive between two classes, but in the case of multi-dimensional class separations, it is called a hyperplane (not a line). When classes are not linearly separable, complexity exists through complex boundaries called kernel tricks on non-linearization. Specifically, the input training dataset, $\left\{x_{i}\right\}i{=}_{1}^{n},$ was transformed to feature space using a non-linear function, φ. The later transformed data obtained in the feature space, $\left\{\varphi (x_{i})\right\}i{=}_{1}^{n},$ underwent a non-linear kernel trick as depicted in Figure 1. When multiple hyperplanes existed, we selected the hyperplane with the best max margin. Researchers have proposed many kernels with relevance to their experiments. Among a few, the most commonly used ones are linear, polynomial, sigmoidal, Gaussian, and exponential RBFs [18]. The selection among the above-mentioned kernels was made based on three performance features. The features applied for the performance measurements were complexity, optimality, and accuracy. The measured data of these features are rated accordingly in Table 2. The initial choice made was the RBF, as it seemed to lead the performance ratings specified in Table 2. The RBFs have high prediction accuracy and optimality, and the complexity measure is quite high for both linear and polynomial kernels, as they involve numerical difficulties when computing the kernel inner products for the featured vectors.

## 4. Proposed Model: RBF-Refined SVM Model

### 4.1. Overview: SVM

**Figure 2:**
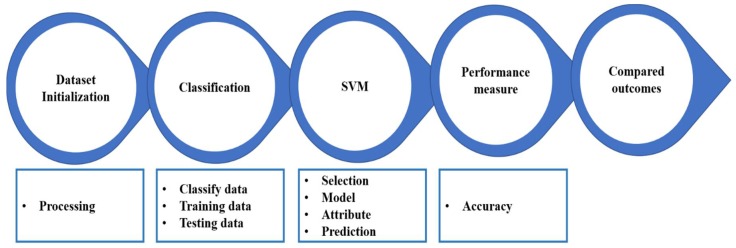
Action stream architecture for the vehicular ad hoc network (VANET) prediction.

This model encompasses four major stages. The preliminary stage is the action stream, comprising dataset initialization. Subsequently, after ensuring the appropriate format of the data for further deployment, it proceeds to the next stage. At the classification stage, the division between training and testing data is fixed and advances into the support vector machine phase. At this stage, appropriate attribute and model selections are made to guarantee an accurate prediction of the test data. The expected outcomes of the prediction of the test data are measured in the penultimate stage, and finally are compared with the outcomes of other techniques, as depicted in Figure 2.

### 4.2. Pre-Processing: Data Initialization

The HETVNET’s traffic dataset from CRAWDAD was transformed to an SVM format for training and testing. Each instance or observation within the dataset was represented as a vector of real numbers. In our dataset, there were few categorical features or attributes. The categorical features and attributes were converted to numerical data. For example, a packet receiving a categorical feature, such as {Yes, No}, was represented as (1, 0). It was also recommended by some researchers to scale the data before application to the SVM. Scaling the numerical range of values helps to avoid complex numerical computations, as the kernels rely on the products of the vector features. In this paper, we did not scale our traffic data, as the usage of linear or polynomial kernels was exempted from this dataset. The usage of linear and polynomial kernels can demonstrate various difficulties in numerical calculations. Henceforth, the transformed SVM-formatted data endured its path forward in making the right choice of model, prior to its application to the SVM.

### 4.3. Classification: Training and Testing Data

At any point in time, each observation of the captured HETVNET traffic had many features and one class label, referred to as the target variable. The goal of the SVM was to produce a model that could predict the target variable’s value for the test data, based on the training dataset. In our investigation, two different approaches of classification patterns were deployed. Initially, the traditional classification task pattern was applied to divide the traffic data into training and testing datasets. Henceforth, the traffic data was divided accordingly: laps one to three were used for training, and laps four and five were used for testing. The traffic dataset classification was done for both north- and southbound directions.

The classification was applied using RBF kernel tuning parameters (C, γ) to achieve the best rate of prediction accuracy. Here, it was noted that the prediction accuracy was directly proportional to the classification procedure/pattern being used. Identifying the perfect (C, γ) parameters may lead to overfitting in certain cases. To avoid issues of overfitting and to get a better prediction accuracy of the test data, we proposed a cross-validation procedure instead of our traditional classification task pattern. The traditional classification pattern was hence twisted further, so as to improve the prediction accuracy. The improved version of classification was termed the cross-validation procedure.

The cross-validation classification pattern involved both training and testing sets, unlike the traditional classifier. The variation was that the training set was kept intact, while the testing set was divided into *n* subsets of equal size. This was done to ensure cross-validation accuracy, where one subset was tested over the other *n − 1* subsets. Researchers propose cross-validation when the available data are limited. In our case, the dataset was not big enough, and hence, may produce a lot of variation in performance estimation. As a result, cross-validation classification was recommended as it reduced the variance by averaging, and the performance remained unaffected due to partition of the data. This pattern of classification also guaranteed the prevention of overfitting of the testing data.

### 4.4. SVM-Based Selection: Model—RBF Kernel

The trial traces of the traffic were mapped in the feature space for class Y (where Y denotes yes and confirms that the packets arrived at the receiver’s end) in a specific area. Correspondingly, the trial traces of the traffic for class N (where N denotes no and assures that the packets did not arrive at the receiver’s end) were mapped in a different area. In the RBF kernel, the entire trace of the traffic was later mapped onto the surface of the hypersphere. The cosine values indicated the similarities between the traces. As the cosine value approached 1, the traces became more similar in the feature space. When the cosine value approached 0, the traces became more dissimilar in the feature space. When the RBF kernel function (*k*) values approached 1 for samples *i* and *j*, this signified that they were in the same class as those denoted in Equation (1). Similarly, when the kernel values approached 0, this signified that they were in different classes, and σ was approximated to 0, as denoted in Equation (2). Hence, ∅ and σ could be adjusted to approach 1, so as to yield better results in our scenario.

(1) $$K\left(\left\{x_{i}\right\}i{=}_{1}^{n},\;\left\{x_{j}\right\}j{=}_{1}^{n},\sigma \right)\;\approx 1$$

(2) $$K\left(\left\{x_{i}\right\}i{=}_{1}^{n},\;\left\{x_{j}\right\}j{=}_{1}^{n},\sigma \right)\;\approx 0$$

The complexity in polynomial kernels is also influenced by the number of hyperparameters, whereas in RBF kernels, this influence is considerably less. Moving forward, RBF kernels were chosen as the ultimate model for our research, due to a better optimality rating through fewer numerical difficulties, perfect accuracy which avoided overfitting, and fewer hyperparameters through the reduction of complexity. Other researchers also justified the use of RBF kernels by their recommendation of datasets that do not have a large number of features. The traffic dataset did not involve a large number of features, and therefore, RBF kernels were used.

### 4.5. Selection: Attribute

The correlation feature evaluator evaluated and ranked the following sixteen features/attributes, as shown in Table 3, for selection: packet-sequence-number (#), time (s), bytes sent, sender latitude, sender longitude, sender speed (km/h), sender altitude (m), receiver latitude, receiver longitude, receiver speed, receiver altitude (m), packet received (Y/N), bytes received, signal strength, and noise strength. The ranking score was based on correlation with the attribute used to predict the class type, which confirmed whether the packets were received or not, with a Y/N or Boolean value. The ranking of attributes were ordered from maximum to minimum, based on their importance. The merit/ranking score ranged from 1 to 0, and was high (close to 1) in cases of certain attributes, as they had a higher correlation/association with the dependent variable. The choice of attribute usage was based on the ranking score, and attributes were given weight based on them predicting the dependent variable accordingly. The highest prioritized attribute, number of bytes received, was ranked with a merit score of 1 (strongly associated), and the next in priority was the signal strength attribute, with a score of 0.999 (strong association). Consequently, we had the noise strength attribute with a score of 0.982, and following this, an eventual decrease to 0 (weak association). The last three attributes in Table 3 could almost be totally neglected and were considered to be of no use. The use of these attributes in the prediction yielded no improvement in the outcomes.

### 4.6. Performance Measure

Performance of the SVMs was measured based on the accuracy percentage, precision, or the number of positively predicted values, and sensitivity percentage of the relevant fractional values, otherwise termed “recall”. Actual results in the prediction fell under the four categories true positive (TP), true negative (TN), false negative (FN), and false positive (FP), which were based on the confusion matrix, as described in Table 4. The confusion matrix was a way to show performance measures of classification models on the collection of test data. Confusion matrices consisted of the information given by classification models about predicted and actual classifications. When an event was positive and predicted positive, then it was deemed a true positive, while if predicted negative, was termed a false negative. Similarly, when an event was negative and predicted positive, then it was a false positive, and otherwise, termed a true negative.

The calculations of precision and recall were made based on the true positive and false positive/negative values accordingly, as shown in Equations (3) and (4). The *F* measure gave the harmonic average of precision and recall, as shown in Equation (5). Matthew’s correlation coefficient (MCC) measures the acute accuracy quality by means of correlation. MCC is a measure which is relied upon as an accuracy rating; however, precision and recall do not consider the true negative values, as shown in Equation (6).

(3) $$Precision=\;\frac{TP}{TP+FP}$$

(4) $$Recall=\;\frac{TP}{TP+FN}$$

(5) $$F=\;\frac{2\times \left(Precision\times Recall\right)}{\left(Precision+recall\right)}$$

(6) $$MCC=\;\frac{\left(TP\times TN\right)-\left(FP\times FN\right)}{\sqrt{\left(TP+FP\right)\left(TP+FN\right)\left(TN+FN\right)\left(TN+FP\right)}}$$

## 5. Experimental Results

### 5.1. Overview of Dataset

This paper used a HETVNET traffic dataset from a Cambridge University repository (CRAWDAD). The traffic used for this research paper focused on the vehicle to road station unit traffic (V2RSU). The traffic was segregated into laps numbered one to five, and were separated accordingly into both northbound and southbound. Senders traveled in a loop between exit 250 and exit 255, and data were collected over five laps. The sender’s car had an external antenna above the front passenger seat, and the receivers were located at the Peachtree Battle bridge (the RSU). The direction of movement in this environment was made up of northbound (exit 250 to exit 255), and southbound (exit 255 to exit 250).

### 5.2. Training and Testing of Dataset

**Figure 3:**
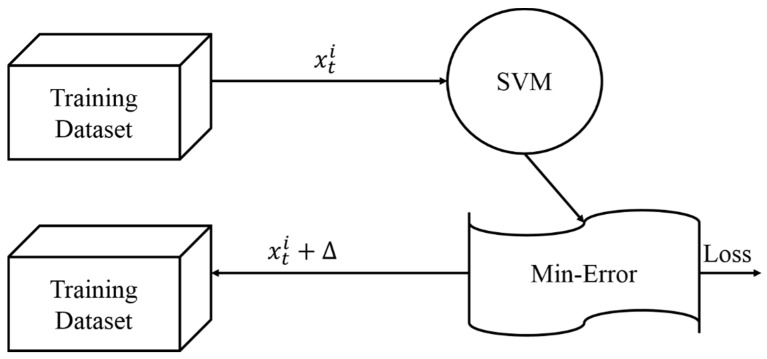
Training and testing architecture.

During training, the SVM uses the data to learn and predict the same behavior for the future. The goal was to give necessary training data ($x_{t}^{i}$) for every observation (*i*) at time (*t*) on traffic of HETVNETs, to predict whether or not the packets were received, by $x_{t}^{i}$ + ∆, as depicted in Figure 3. Using the induction learning technique, it withheld two stage-processing agendas. During the initial stage of the training phase, the SVM acquired knowledge from the training set. In the later stage, the testing phase, it evaluated and associated the members with their respective classes accordingly.

### 5.3. Comparison of Outcomes

In this paper, the dataset had sixteen attributes, and the class prediction was based on whether or not the packets were received, with a YES or NO, represented by Y/N. The training dataset’s number of instances was 10,000, and the test dataset’s number of instances was 6346. In order to compare and demonstrate the performance of non-parametric and parametric classifiers, an ideal choice of logistic regression was made, as the dependent/predictor variable was categorical. The outcome of classes receiving the packet was categorized with Y/N. Complete accuracy measures of parametric (logistic regression) and non-parametric classifiers (SVM types) are listed below in Table 5. The average accuracy percentage of logistic regression (LR), a parametric classifier, was 66%, while LIBLINEAR was below 65%. LIBSVM seemed to be much better than LIBLINEAR and LR (parametric classifier); however, the SVM with an RBF kernel under a grid search was a well-refined and optimal package, trained to solve quadratic programming problems while ensuring an accuracy percentage of over 99%. A comparison of the results is provided in Table 5.

This illustrates that when the cross-validation method was applied to the selected SVMs and logistic regression [18,19,20,21], the accuracy of LIBSVM and LIBLINEAR improved significantly more than that of the SVM-RBF. The reason for this was that they were run over a simple probability, in an effort to further optimize and enhance performance, by tuning the cost (C) parameter. When cross-validation was applied to them, the performance elevated and also resulted in bad generalization/overfitting. The SVM-RBF was already a fine-tuned technique, embedded with the advanced probability techniques of SVMs. This technique jointly optimized cost and gamma parameters. The SVM-RBF guaranteed a consistently good generalization with no overfitting curves when the variance was averaged by the cross-validation method.

**Figure 4:**
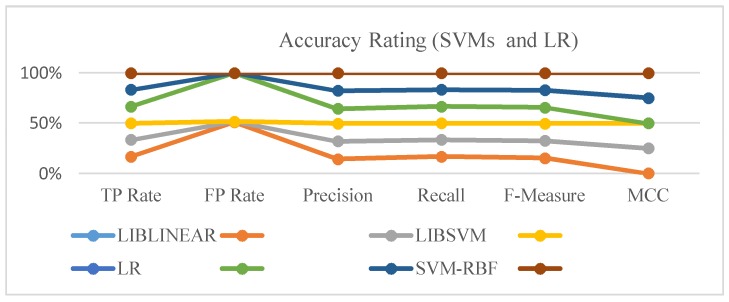
Accuracy rating (SVMs and LR).

The accuracy of the dataset for lap five of the northbound traffic is showcased by class in Table 6, where Y indicates that the packets were received, while N denotes that the packets were not received. The dataset had 16 features/attributes and 6346 instances upon which the test run was done, to showcase the compared performance measures of LIBLINEAR, LIBSVM, and the SVM-RBF. TP reflects the true positive rate, while FP reflects the false positive rate. Receiver operating characteristics (ROCs) had an area ranging from zero to one. When the ROC area [22] value was less than 0.500, the prediction was labeled as random/poor. On the other hand, it was considered a good, excellent, or perfect prediction as the value approached 1.000. Precision and recall were computed to confirm the accuracy rate with *F*-measure, which expressed the stability between them. Precision–recall curves (PRCs) highlighted the differences between models. Finally, Matthew’s correlation coefficient (MCC) was used as a geometric mean, which measured the quality of classification. All accuracy measures are explicitly represented in Figure 4, which showcases that the accuracy rating of the SVM-RBF outperformed the logistic regression classifier and other SVMs.

## 6. Conclusions and Future Work

In this paper, the importance of classifying the dataset for testing and training was made clear. The mapping with an RBF kernel in a grid search revealed more promising outcomes than those of traditional SVMs. The refinement of traditional SVMs, with a suitable choice of RBF-induced kernels in a grid search, minimized computation time and delivered a swift SVM. The performance of the SVM-RBF’s outcomes was noticeably superior to that of LIBSVM’s and LIBLINEAR’s. The SVM is a strong candidate in the field of machine learning, which, when tuned appropriately, can yield more promising results in the future. This strategy can be benchmarked in future prediction services using big datasets. In the near future, a bigger dataset can be used for a test run of the SVMs to reveal the outstanding scalability and efficiency of the SVM-RBF in comparison with other SVM combinations, such as the maximum likelihood classifier (SVM-MLC). Additionally, a routing protocol for HETVNETs will be developed, so as to use results of the proposed prediction model and overcome problems such as broadcasting storms and intermittent connectivity.

## Figures and Tables

**Table 1:** Kernel functions in support vector machines (SVMs).

SVM Type	Kernel Function $K(x_{i,}y$)
Linear	$x_{i}^{T}y$
Polynomial	($\gamma x_{i}^{T}y+r)$^d^
Sigmoid	tanh($\gamma x_{i}^{T}y+r)$
Radial Basis Function (RBF)	exp (-γ‖$x_{i}-\;x_{j}$‖^2^, γ>0

**Table 2:** Performance measures of SVM kernels.

Kernel	Complexity	Optimality	Accuracy
Linear	High	High	Medium
Polynomial	High	Medium	Medium
RBF	Low	High	High
Sigmoidal	Medium	Low	Medium

**Table 3:** Attribute ranking score card.

Average-Merit	Average-Ranking Score	Attribute #/Name
1 ± 0	1 ± 0	14 Bytes Received
0.999 ± 0	2 ± 0	15 Signal Strength
0.982 ± 0	3 ± 0	16 Noise Strength
0.474 ± 0.003	4 ± 0	8 Sender Altitude (m)
0.468 ± 0.002	5 ± 0	7 Sender Speed (km/h)
0.415 ± 0.003	6 ± 0	12 Receiver Altitude (m)
0.336 ± 0.001	7 ±0	1 Packet-Sequence-No #
0.335 ± 0.004	8 ± 0	2 Time in Sec
0.104 ± 0.001	9 ± 0	9 Receiver Latitude
0.091 ± 0.002	10 ± 0	10 Receiver Longitude
0.078 ± 0.001	11 ±0	6 Sender Longitude
0.078 ± 0.001	12 ± 0	5 Sender Latitude
0.017 ± 0.003	13 ± 0	3 Time in unit sec
0 ± 0	14 ± 0	4 Bytes Sent
0 ± 0	15 ± 0	11 Receiver Speed

**Table 4:** Confusion matrix. FN: false negative; false positive; TN: true negative; TP: true positive.

Actual/Predicted	No	Yes
**No**	TN	FP
**Yes**	FN	TP

**Table 5:** Accuracy measures among SVMs. “N” and “S” in the Traffic Dataset column indicate northbound and southbound directions, respectively.

Traffic Dataset	Using Traditional Training Set				Using Cross-Validation Set			
	Accuracy (SVM-RBF) %	Accuracy (LIBSVM) %	Accuracy (LIBLINEAR) %	Logistic Regression (LR) %	Accuracy (SVM-RBF) %	Accuracy (LIBSVM) %	Accuracy (LIBLINEAR) %	Logistic Regression (LR)%
Lap5 N	99.8	94.7	44.2	62.3	100	99.7	78.6	82.5
Lap4 N	98.7	93.5	63.1	66.5	99.7	97.3	84.3	86.6
Lap3 N	97.5	89.5	61.5	65.1	99.8	93.7	87.6	85.1
Lap2 N	99.1	92.7	57.8	59.5	99.9	98.7	89.7	79.3
Lap1 N	96.7	92.5	67.1	77.4	99.9	98.5	94.3	89.6
Lap5 S	96.5	90.5	65.5	64.5	99.8	97.7	88.6	85.4
Lap4 S	98.3	93.5	63.1	66.3	99.7	97.3	83.4	89.3
Lap3 S	95.5	85.5	61.5	63.5	99.8	94.7	86.5	83.6
Lap2 S	99.1	92.7	57.8	67.9	99.9	98.7	89.7	89.7
Lap1 S	96.7	92.5	67.1	66.3	99.9	98.5	94.3	88.4

**Table 6:** Accuracy by class: packets received (yes (Y)/no (N)). LR: logistic regression; MCC: Matthew’s correlation coefficient; PRC: precision–recall curve; ROC: receiver operating characteristic.

Classifier Type	TP Rate	FP Rate	Precision	Recall	*F*-Measure	MCC	ROC Area	PRC Area	Class
LIBLINEAR	1.000	1.000	0.787	1.000	0.881	0.000	0.500	0.787	Y
	0.000	0.000	0.000	0.000	0.000	0.000	0.500	0.213	N
LIBSVM	1.000	0.011	0.997	1.000	0.999	0.993	0.995	0.997	Y
	0.989	0.000	1.000	0.989	0.995	0.993	0.995	0.991	N
SVM-RBF	1.000	0.000	1.000	1.000	1.000	1.000	1.000	1.000	Y
	1.000	0.000	1.000	1.000	1.000	1.000	1.000	1.000	N
LR	1.000	0.951	0.822	1.000	0.924	0.000	0.650	0.822	Y
	0.000	0.000	0.000	0.000	0.000	0.000	0.585	0.265	N
